# Hawk tea (*Litsea coreana* Levl. var. *lanuginose*) attenuates CCl_4_-induced hepatic damage in Sprague-Dawley rats

**DOI:** 10.3892/etm.2012.840

**Published:** 2012-11-28

**Authors:** XIN ZHAO

**Affiliations:** Department of Biological and Chemical Engineering, Chongqing University of Education, Chongqing 400067, P.R. China

**Keywords:** Hawk tea, hepatic damage, CCl_4_, cytokines, inflammation

## Abstract

Hawk tea (*Litsea coreana* Levl. var. *lanuginose*) is a traditional Chinese drink similar to green tea. In the present study, the preventive effects of Hawk tea on hepatic damage induced by carbon tetrachloride (CCl_4_) were studied in Sprague-Dawley rats. Silymarin was used as a positive control. Hawk tea was successfully shown to prevent hepatic damage in the rats. Serum levels of AST, ALT and LDH were significantly decreased when the rats were treated with varying concentrations of Hawk tea compared with silymarin (P<0.05). The lowest enzyme activities were exhibited in the 400 mg/kg Hawk tea group. This group showed reduced levels of the serum proinflammatory cytokines IL-6, IFN-γ and TNF-α. In particular, the IFN-γ level decreased markedly compared with the other concentration groups. The histopathology sections of liver tissue in the 400 mg/kg Hawk tea group recovered well from the CCl_4_ damage, but the sections of the other concentration groups showed necrosis to a more serious degree. Reverse transcription-polymerase chain reaction (RT-PCR) and western blot analyses of the inflammation-related genes iNOS, COX-2, TNF-α and IL-1β in the rat livers were tested. The 400 mg/kg Hawk tea group showed significantly decreased mRNA and protein expression levels of iNOS, COX-2, TNF-α and IL-1β compared with the control group. Accordingly, 400 mg/kg Hawk tea potentially contributes to the prevention of CCl_4_-induced hepatic damage *in vivo*. A 200 or 100 mg/kg dose of Hawk tea also demonstrated preventive effects against hepatic damage.

## Introduction

Hawk tea is a beverage with a long history of use in southwest China, particularly in Chongqing, Sichuan and Guizhou (1). The utilization of Hawk tea has surpassed that of green tea for a long time. Hawk tea is made from a plant that belongs to the *Litsea coreana* Levl. var., its full scientific name being *Litsea coreana* Levl. var. *lanuginose*(2). *Litsea coreana* leve is a traditional Chinese medicine which has been recorded in ‘Ben Cao Gang Mu’ (the Compendium of Materia Medica, a classical traditional Chinese medicine book) that has traditionally been used as a hypolipidemic drug in southern China for hundreds of years (3). Certain studies have demonstrated that Hawk tea possesses antioxidant, hyperglycemic, hypolipemic and anti-inflammatory effects (1).

Numerous lines of evidence have suggested oxidative stress and inflammation in the etiology of liver disease, cardiovascular disease and cancer (4,5). As a result, carbon tetrachloride (CCl_4_), which produces reactive-free radicals when metabolized, has been widely used as a solvent to develop hepatic damage in animal models (6). CCl_4_ is able to increase lipid peroxidation on the cell membrane and alter enzyme activity, thereby inducing hepatic injury and necrosis (7).

The release of aspartate aminotransferase (AST) and alanine aminotransferase (ALT) into the blood has been shown to increase as cell membranes of injured or dying hepatocytes lose their integrity (8). AST is released into the serum in proportion to cell damage and is most elevated in the acute phase of cell necrosis. ALT is released early in liver damage and remains elevated (9). Lactate dehydrogenase (LDH) is of greatest use in monitoring liver injury disorders (10). Cytokines, including IL-6, IFN-γ and TNF-α, are small proteins that are produced and released from numerous cells under certain physiological and pathological conditions. Cytokines may be centralized around this organ as it hosts cells that are highly susceptible to the action of the proteins (11). Unlike sustained hepatocellular damage, acute hepatic damage is temporary and ends with the return of normal liver histology and function. A stress situation may be induced in hepatocytes by hypatotoxins with a subsequent release of chemokines followed by the accumulation of inflammatory cells and hepatocellular damage (12). Blood assays of ALT, AST, LDH and cytokines are an important standard measure of the hepatic damage levels.

The iNOS, COX-2, IL-lβ and TNF-α genes are responsible for the hepatic damage and deleterious effects in the liver caused as by a response to inflammatory stimuli (13,14).

In the present study, the *in vivo* anti-inflammatory effects of Hawk tea were investigated. Serum assays of AST, ALT, LDH, TG (triglyceride), TC (total cholesterol) levels and inflammation-related cytokines and examinations of liver tissue histology and inflammation-related gene expression were used to determine the preventative effects of Hawk tea against CCl_4_-induced hepatic damage in Sprague-Dawley (SD) rats.

## Materials and methods

### Preparations of Hawk tea

Hawk tea was purchased in a local market (Yuzhong District, Chongqing City, China) and stored at −80°C prior to being freeze-dried to produce a powder. A 20-fold volume of methanol was added to the powdered samples and then extracted twice by stirring overnight. The methanol extract was evaporated using a rotary evaporator (N-1100; Eywla, Tokyo, Japan), concentrated and then dissolved in dimethyl sulfoxide (DMSO; Amresco, Solon, OH, USA) to adjust to the stock concentration (20%, w/v).

### Induction of hepatic damage

Male SD rats (n=60, 7-weeks-old) were purchased from the Experimental Animal Center of Chongqing Medical University (Chongqing, China). The animals were maintained in a temperature controlled facility (temperature 25±2°C, relative humidity 50±5%) with a 12-h light/dark cycle and free access to a standard rat diet and water.

To investigate the preventive effects of Hawk tea against CCl_4_-induced hepatic damage, the animals were divided into six groups consisting of 10 rats each. The experimental design was as follows: the normal control group were administered with distilled water for 14 days and a single dose of vehicle [2 ml/kg body weight (bw) olive oil, p.o.], while the CCl_4_ control group received a 14-day repeated oral administration of distilled water followed by a single administration of CCl_4_ (2 ml/kg bw dissolved in olive oil, 1:1, v/v) on the last day to induce liver damage. The three Hawk tea + CCl_4_ groups received 100, 200 or 400 mg/kg bw of Hawk tea and the positive control group received 2 weeks of 100 mg/kg bw silymarin dissolved in water, with hepatic damage induced in the same manner as that mentioned above. The rats were anesthetized 24 h after the administration of CCl_4_ and sacrificed using CO_2_(15). Blood and livers were collected and preserved at −70°C until biological assays were performed. These experiments followed a protocol approved by the Animal Ethics Committee of Chongqing Medical University (Chongqing, China).

### AST, ALT and LDH levels in serum

The AST and ALT levels of the serum were determined using commercially available kits (Shanghai Institute of Biological Products Co., Ltd., Shanghai, China). LDH levels of the serum were determined using specific commercially available kits (Cayman Chemical Company, Ann Arbor, MI, USA).

### ELISA analysis of inflammation-related cytokines in serum

For the serum cytokine assays, blood from the inferior vena cava was collected into tubes and centrifuged (3,000 rpm, 10 min, 4°C). The serum was then aspirated. The serum concentrations of the inflammatory-related cytokines IL-6, IFN-γ and TNF-α (Biolegend, San Diego, CA, USA) were measured by ELISA according to the manufacturer’s protocol. Briefly, following the addition of the biotinylated antibody reagent to the 96-well plates, the supernatants of the homogenized serum were incubated at 37°C in CO_2_ for 2 h. Subsequent to washing with PBS, streptavidin-peroxidase (HRP) solution was added and the plate was incubated for 30 min at room temperature. The absorbance was measured at 450 nm using a microplate reader (16).

### Histological examination of the liver tissue

At the end of the experimental period, the liver tissues were removed and cleaned in saline to remove any residue. For the histological investigations, the liver tissues were fixed in 10% (v/v) buffered formalin for 24 h, cut into 2 longitudinal halves and then embedded into paraffin. Paraffin sections 4 *μ*m-thick were stained with hematoxylin and eosin (H&E) prior to microscopic observation (BX41, Olympus, Tokyo, Japan) (17).

### Reverse transcription-polymerase chain reaction (RT-PCR) of inflammation-related gene expressions in the liver tissue

Total RNA was isolated from the liver tissue using TRIzol reagent (Invitrogen, Carlsbad, CA, USA) according to the manufacturer’s recommendations. The RNA was digested with RNase-free DNase (Roche, Basel, Switzerland) for 15 min at 37°C and purified using a RNeasy kit (Qiagen, Hilden, Germany) according to the manufacturer’s instructions. cDNA was synthesized from 2 *μ*g total RNA through incubation at 37°C for l h with AMV reverse transcriptase (GE Healthcare, Little Chalfont, UK) and random hexanucleotides, according to the manufacturer’s instructions.

The primers used to specifically amplify the genes of interest were: iNOS forward: 5′-AGA GAG ATC GGG TTC ACA-3′ and reverse: 5′-CAC AGA ACT GAG GGT ACA-3′; COX-2 forward: 5′-TTA AAA TGA GAT TGT CCG AA-3′ and reverse: 5′-AGA TCA CCT CTG CCT GAG TA-3′; TNF-α forward: 5′-GAC CCT CAG ACT CAG ATC ATC CTT CT-3′ and reverse: 5′-ACG CTG GCT CAG CCA CTC-3′; IL-1β forward: 5′-CTC CAT GAG CTT TGT ACA AGG-3′ and reverse: 5′-TGC TGA TGT ACC AGT TGG GG-3′. The internal control gene of GAPDH was amplified using the primers: forward: 5′-CGG AGT CAA CGG ATT TGG TC-3′ and reverse: 5′-AGC CTT CTC CAT GGT CGT GA-3′. Amplification was performed in a thermal cycler (Eppendorf, Hamburg, Germany) with cycles of denaturation. The amplified PCR products were run in 1.0% agarose gels and visualized by ethidium bromide (EtBr) staining (18).

### Protein extraction and western blot analysis in the liver tissue

Total liver tissue protein was obtained with RIPA buffer as described by Kim *et al*(19). Protein concentrations were determined with a Bio-Rad protein assay kit (Hercules, CA, USA). For the western blot analysis, aliquots of the lysate containing 30–50 *μ*g protein were separated by sodium dodecyl sulfate-polyacrylamide gel electrophoresis (SDS-PAGE) and then electrotransferred onto a nitrocellulose membrane (Schleicher and Schuell, Keene, NH, USA). The membranes were subjected to immunoblot analysis and the proteins were visualized by an enhanced chemiluminescence (ECL) method (GE Healthcare). The cell lysates were separated by 12% SDS-PAGE, transferred onto a polyvinylidene fluoride membrane (GE Healthcare), blocked with 5% skimmed milk and hybridized with primary antibodies (diluted 1:1,000). The antibodies against iNOS, COX-2, TNF-α and IL-1β were obtained from Santa Cruz Biotechnology Inc. (Santa Cruz, CA, USA), then incubated with the horseradish peroxidase-conjugated secondary antibody (Santa Cruz Biotechnology Inc.) for 1 h at room temperature. The blots were washed three times with PBS-T and then developed by enhanced chemiluminescence (Amersham Life Science, Arlington Heights, IL, USA).

### Statistical analysis

Data were presented as the mean ± SD. Differences between the mean values for individual groups were assessed using a one-way ANOVA with Duncan’s multiple range test. P<0.05 was considered to indicate a statistically significant difference. SAS version 9.1 (SAS Institute Inc., Cary, NC, USA) was used for statistical analyses.

## Results

### Effect of Hawk tea on serum levels of AST, ALT and LDH

**Figure 1.**
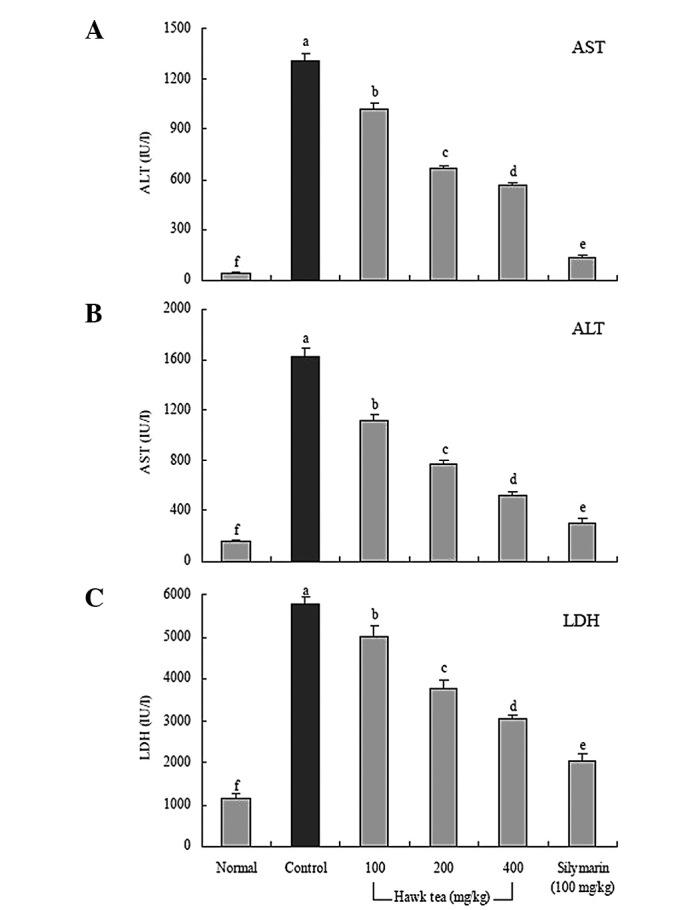
Effect of Hawk tea on serum levels of (A) aspartate aminotransferase (AST), (B) alanine aminotransferase (ALT) and (C) lactate dehydrogenase (LDH) in CCl_4_-induced hepatic damage. (a–f) Mean values with different letters over the bars represent significant differences (p<0.05) according to Duncan’s multiple range test.

The AST level of normal rats was 155.5±15.6 IU/l, however, the level in the CCl_4_ control rats was significantly increased to 1623.7±67.6 IU/l. The levels of AST in the 100, 200 or 400 mg/kg Hawk tea groups were 1121.3±44.2, 765.6±32.3 and 524.6±31.2 IU/l, respectively. The level of AST in rats treated with silymarin (100 mg/kg) decreased to 303.0±37.3 IU/l, which was lower than the level in rats treated with Hawk tea (Fig. 1A).

The ALT level in the normal group was 43.5±6.5 IU/l, whereas that of the control group was 1308.8±44.2 IU/l, reflecting a marked increase. The ALT levels in the 100 and 200 mg/kg Hawk tea groups decreased to 1024.5±31.2 and 667.8±18.5 IU/l, respectively. A high concentration (400 mg/kg) of Hawk tea treatment resulted in a further decreased ALT level (570.6±14.5; Fig. 1B). Thus, from the results of serum levels of AST and ALT, it was confirmed that Hawk tea generally inhibited hepatic damage induced by CCl_4_, although the 400 mg/kg Hawk tea group demonstrated this to the best effect.

The levels of LDH in the 100 and 200 mg/kg Hawk tea groups were 5000.1±258.6 and 3785.2±179.5 IU/l, respectively, which were slightly lower than the level of the control group (5784.2±145.6 IU/l). However, LDH levels in the 400 mg/kg and silymarin groups were 3033.6±88.8 and 2023.6±194.5 IU/l, respectively, whereas the normal group showed the lowest level at 1155.8±98.6 IU/l (Fig. 1C). The LDH levels in the Hawk tea groups were lower than those of the control group, similar to the observations of the AST and ALT levels.

### Effect of Hawk tea on serum levels of IL-6, IFN-γ and TNF-α

**Figure 2.**
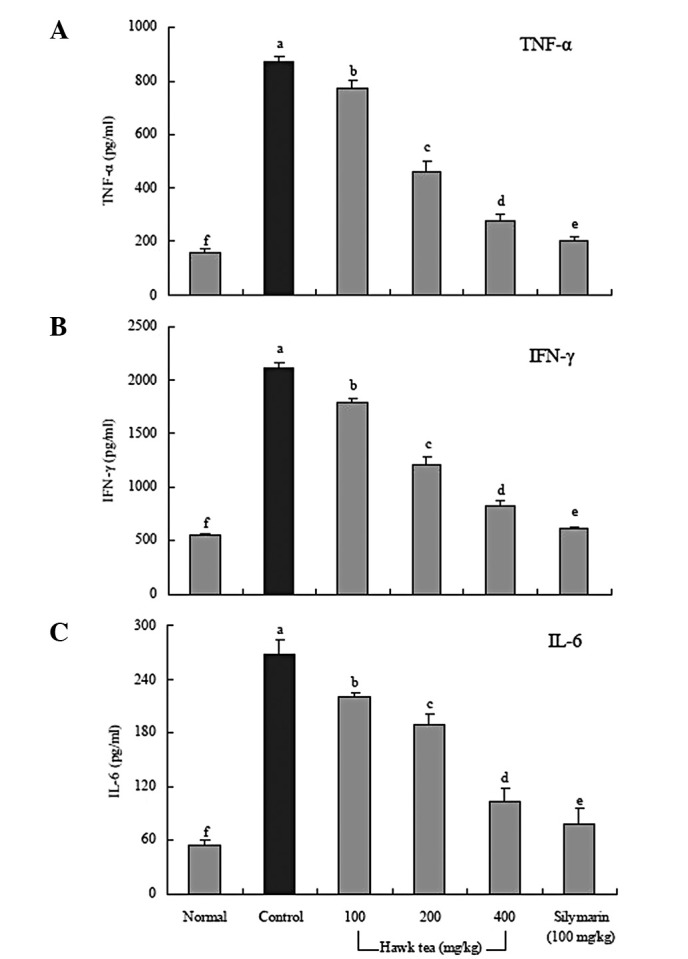
Effect of Hawk tea on serum levels in (A) IL-6, (B) IFN-γ and (C) TNF-α levels in CCl4-induced hepatic damage. (a–f) Mean values with different letters over the bars represent significant differences (p<0.05) according to Duncan’s multiple range test.

Serum IL-6, IFN-γ and TNF-α levels in rats in the 100 and 200 mg/kg Hawk tea-treated groups were significantly lower than those of the control group (Fig. 2). The reductions observed in the IL-6 and IFN-γ levels of the 400 mg/kg Hawk tea group were 78.0% and 82.4%, respectively, compared with the control group. The TNF-α level in rats treated with 400 mg/kg Hawk tea also decreased by 83.8%. However, the levels of these proinflammatory cytokines in rats treated with silymarin were similar to those of rats in the normal group.

### Effect on the liver tissues in rats with CCl_4_-induced hepatic damage

**Figure 3.**
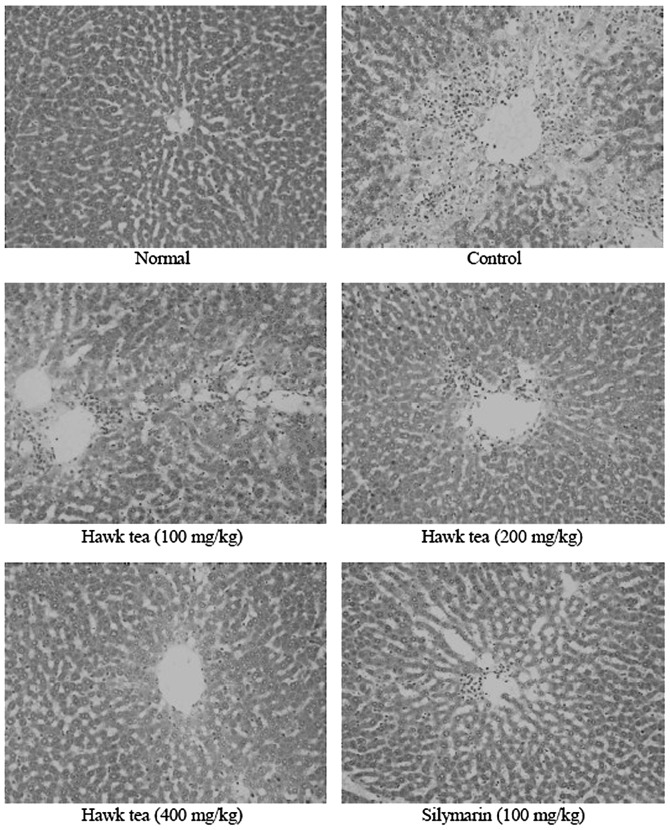
Histological images of the liver tissues in rats with CCl_4_-induced hepatic damage (magnification, ×200).

The sections from the mice in the control group showed widespread areas of congestion and hemorrhages in the centrilobular zone, as well as necrosis involving all the hepatocytes in the centrilobular zone (moderate grade 3). There was also bridging of the areas of necrosis (Fig. 3). The sections from the silymarin (100 mg/kg)-treated group did not show any evident hepatic damage, but were also different from normal (moderate grade 0). The 100 mg/kg Hawk tea group showed moderate congestion and hemorrhages in the area around the centrilobular vein and extending into the midzonal cells (mild grade 2), with the majority of lobules being affected. Areas of confluent necrosis were limited to the liver cells surrounding the centrilobular vein. The tissue sections of the 200 and 400 mg/kg Hawk tea groups appeared less like normal tissue (grade 1). The livers showed minimal congestion and necrosis of single hepatocytes limited to the area immediately around the centrilobular vein, with many of the lobules not being affected. The 200 mg/kg Hawk tea group showed mild inflammatory infiltration, whereas the 400 mg/kg Hawk tea group did not show any inflammatory infiltration. These results demonstrate that a higher concentration of Hawk tea decreased the degree of inflammation.

### Effects of Hawk tea on mRNA and protein expression levels of iNOS, COX-2, TNF-α and IL-1β in the liver tissues of rats treated with CCl_4_

**Figure 4.**
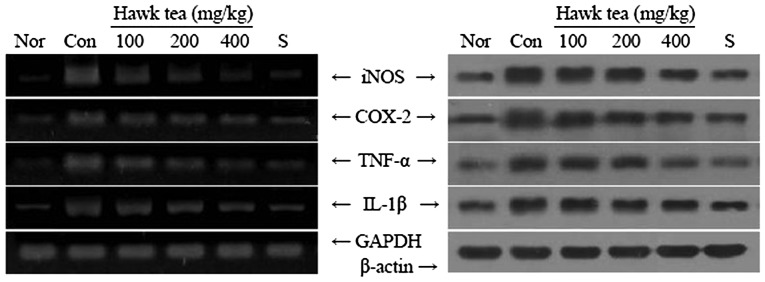
Effects of Hawk tea on mRNA and protein expression levels of iNOS, COX-2, TNF-α and IL-1β in the liver tissues of rats treated with CCl4. Nor, Normal; Con, Control; S, Silymarin (100 mg/kg).

RT-PCR and western blot analyses were conducted to investigate whether the inhibitory effect of the Hawk tea on inflammation was due to transcriptional regulation of inflammatory mediators in the liver, including iNOS, COX-2, TNF-α and IL-1β. CCl_4_ treatment significantly increased the mRNA and protein levels of these inflammatory mediators (Fig. 4). However, 400 mg/kg Hawk tea significantly decreased the mRNA and protein expression levels of iNOS, COX-2, TNF-α and IL-1β, similar to the silymarin group (P<0.05). Liver tissues from the 100 and 200 mg/kg Hawk tea-treated rats showed a decreased expression of these genes compared with the control group.

## Discussion

AST and ALT are enzymes located in liver cells that leak out into the general circulation when the cells are injured. AST is located in many body tissues, including the heart, muscle, kidney, brain and lung tissues. ALT is located predominately in the liver, with lesser quantities located in the kidneys, heart and skeletal muscles (20). LDH is an enzyme located in numerous body tissues, including the liver where elevated levels of LDH may indicate liver damage. AST and ALT levels have also been used to evaluate the hepatoprotective activities of medicinal plants in CCl_4_-induced hepatic damage in animal models (21,22). Raju *et al*(23) reported that the serum AST and ALT levels in CCl_4_-treated rats was markedly increased compared with those of rats in the normal group, which indicated that liver damage was significantly induced by CCl_4_. Tea is a beverage consumed worldwide and regarded as a healthy source of pleasure. Green tea was originally recommended in traditional Chinese medicine and has gained considerable attention due to its preventive effects against CCl_4_-induced hepatic damage (24). Hawk tea and green tea were important beverages in southwest China for hundreds of years. Hawk tea contains a higher content of catechins, minerals and flavonoid compounds (25). Drinking Hawk tea for a long period of time is believed to keep a person mentally and physically healthy. Certain studies have demonstrated that Hawk tea has anti-inflammatory effects (1). Silymarin is a unique flavonoid complex, with both *in vitro* and animal studies suggesting that silymarin possesses hepatoprotective properties that protect liver cells against toxins (26). In the present study, Hawk tea and silymarin had an *in vivo* preventive effect on CCl_4_-induced hepatic damage.

The serum levels of cytokines, including IL-6, TNF-α, IL-1β and IFN-γ, in patients with inflammatory diseases are higher than those in healthy people (27). Thus, lower levels of IL-6, IFN-γ and TNF-α are indicative of improved anti-inflammatory effects. Hawk tea has consequently demonstrated a good protective effect against hepatic damage in studies thus far. Hepatocytes bear a variety of cytokine receptors and the inflammatory cytokines IL-6, IFN-γ and TNF-α play pathogenic roles in liver disease (28). Although systemic IL-6 levels are elevated following traumatic hemorrhage, hepatocellular function is impaired and liver injury occurs (29). IFN-γ increases such injury by stimulating hepatic inflammation and aggravating liver damage (30). TNF-α is also a key mediator in a number of experimental liver injury models (31).

Histopathological analysis is an important clinical standard used to diagnose hepatic damage (32). In addition, the histopathological examination of rat liver sections is reported as an effective method to check the hepatoprotective activity against the CCl_4_-induced hepatic damage in the rat model (33). From the sections examined in the present study, Hawk tea was observed to exert a preventive effect against CCl_4_-induced hepatic damage. Hawk tea exhibits antioxidative effects (1). Compounds with antioxidant properties may also have anti-inflammatory effects and actually prevent the activation of inflammatory signals (34).

The present study demonstrated that Hawk tea was effective in the prevention of CCl_4_-induced hepatic damage in SD rats. The results show that the hepatoprotective effects of Hawk tea may be due to decreased serum levels of AST, ALT, LDH and proinflammatory cytokines, including TNF-α, IL-6 and IFN-γ. Histopathological studies also showed that Hawk tea was able to prevent CCl_4_-induced inflammation in the liver. Furthermore, mRNA and protein expression levels of inflammation-related genes in the liver, including iNOS, COX-2, TNF-α and IL-1β were significantly reduced in the Hawk tea-treated rats (P<0.05). These results suggest that Hawk tea is potentially useful in the treatment or prevention of chemical-induced hepatic damage *in vivo*. The present study demonstrated that Hawk tea was effective in the prevention of CCl_4_-induced hepatic damage in SD rats.
